# A Real-Time Spectroscopic Sensor for Monitoring Laser Welding Processes

**DOI:** 10.3390/s90503376

**Published:** 2009-05-07

**Authors:** Teresa Sibillano, Antonio Ancona, Vincenzo Berardi, Pietro Mario Lugarà

**Affiliations:** 1 CNR-INFM Regional Laboratory LIT^3^, via Amendola 173, Bari, Italy; E-Mails: ancona@fisica.uniba.it (A.A.); v.berardi@poliba.it (V.B.); lugara@fisica.uniba.it (P.L.); 2 Dipartimento Interateneo di Fisica, Università e Politecnico di Bari, Via E. Orabona 4, I-70126 Bari, Italy

**Keywords:** Optical sensor, laser welding, plasma spectroscopy

## Abstract

In this paper we report on the development of a sensor for real time monitoring of laser welding processes based on spectroscopic techniques. The system is based on the acquisition of the optical spectra emitted from the laser generated plasma plume and their use to implement an on-line algorithm for both the calculation of the plasma electron temperature and the analysis of the correlations between selected spectral lines. The sensor has been patented and it is currently available on the market.

## 1. Introduction

Traditional off-line inspection of welded joints is expensive and reduces productivity, and the lack of effective on-line controls in laser machining is one of the main obstacles for the full implementation of laser welding technologies in industrial applications.

Several solutions have been proposed in recent years for the development of automated on-line laser welding monitoring sensors. Spectroscopic investigation of the plasma optical emission provides a number of potential advantages for a detailed analysis of defects as a function of the laser operation parameters and the material properties.

In this work an overview will be given of the recently developed optical-based monitoring systems for laser welding processes. Then, we will focus on our last experimental results on the development of an optical sensor, based on plasma spectroscopy, especially conceived for real-time control and optimization of the welding processes using a CO_2_ laser source.

## 2. Optical sensing for laser welding

In laser welding the laser-metal interaction is usually associated with the ejection of material from the interaction area. The ejected material contains excited atoms and ions and it is commonly named plume. The material moves through the incident beam and is thus further heated to temperatures exceeding the vaporization temperature. Under certain conditions, the overall effect is to produce a rapid increase in the level of ionization within the plume with the formation of a plasma. Because the plasma is created only when vaporization occurs, its presence during laser welding may provide useful information about the welding conditions.

Several signals coming from the plasma can be used to yield information on the possible presence of defects during the process [1]. Among the many possible techniques for the development of optical sensors, the most effective ones are those based on the measurement of the spatially integrated optical intensity by one or more photodiodes as well as the spectroscopic analysis of the UV/VIS emission [2,3]. Such kind of optical sensors based on the plasma plume optical emission have been used successfully in industrial environments for the detection of several types of welding defects.

Several works have reported on the development of optical sensors based on photodiodes as a tool for the detection of the light intensity from the plasma produced during the process. Gaztweiler *et al.* [4] monitored the keyhole plasma by using an array of photodiodes collecting light at different viewing angles with respect to the beam axis. By this arrangement each detector monitors a different region of the plasma. The overall plasma intensity distribution inside the keyhole was then estimated by combining the signals. Such a sensor was reliable in monitoring the penetration depth on thick samples of steel sheets as well as in obtaining information on the bead shape.

Park *et al.* [5,6] monitored both the bead shape and the full penetration by a photodiode-based acquisition of the UV emission from plasma and the IR from spatters. A simultaneous measurement of the spectral line intensities and of the IR radiation from the weld pool gave a possible correlation between the plasma characteristics and the size and temperature of the weld pool. In this way the authors show that simultaneous measurements of the spectral line intensities from the plasma emission, together with IR emission from the weld pool, provide a way to relate changes in plasma characteristics to the size and temperature of the weld pool itself. The limit of this approach is the difficulty to separate the IR emission coming from the weld pool from the IR signal coming from the plasma.

Peters *et al.* [7,8] reported a non-intrusive optical sensing technique for Nd:YAG laser welding based on the simultaneous detection of the light radiated by the plasma plume above the welding surface and of the light back propagating in the cladding of the laser beam delivery fiber (cladding power monitor, CPM). The cladding was coupled to the core of the monitor fiber and then delivered to a UV/VIS photodiode. The system was used to demonstrate the correlation between the optical signals of the plume and laser welding faults. The same group has recently reported [9,10] on a real-time focus control during Nd:YAG laser welding.

Bardin *et al.* [11,12] described the design of a closed-loop system that monitors the focal position to ensure full penetration during Nd:YAG laser welding processes. The focus position monitoring system was based on the chromatic aberration of the focusing optics: the results obtained showed that the spectral analysis of the light emitted from the weld pool detected by three different photodiodes gave information on the focal error.

To optimize the process parameters, other authors have investigated the stability of photodiode signals by monitoring fluctuations. A closed loop control system has been developed by Bagger and Olsen [13] to control the laser power by observing the light emission from the root-side of the sheet. The control system is able to adjust the laser power to keep an even root seams if the welding speed or the thickness are changed. The main drawback of such a system is that it is not suitable for lap welding with a thickness greater than 1.25 mm.

### 2.1. Plasma spectroscopy

In laser welding it is well known that a strong plasma optical emission is observable right above the keyhole and can be easily collected by using optical fibers [14]. As a consequence, plasma plume optical spectroscopy is a very promising technique for realizing a reliable on-line monitoring of the quality of welded joints and in general for keeping under control the welding process. The spectroscopic approach has been easily extended to arc welding by several research groups [15,16], improving the performance of the arc-welding monitoring systems.

Plasma optical spectra are characterized by the presence of emission lines coming both from the excited atoms and from the ions produced during the laser-surface interaction. A careful spectroscopic characterization of such emission lines allows to determine the chemical composition and the dynamics of interaction of the different chemical species inside the plume.

The measurement of the plasma electron temperature as well as the analysis of the plasma optical spectra by using the Covariance Mapping Technique have been the subject of several papers published by our research group over the last few years [17-20]. The final objective was to combine the two above mentioned techniques to develop an optical sensor for real-time defect recognition during industrial laser processes.

The plasma electron temperature can be obtained from the measurement of the relative intensity of a set of spectral lines free from self-absorption and by the application of the Boltzamnn plot method. The intensity I_mn_ of a plasma emission line associated with the decay between levels E_m_ and E_n_ is related to the energy of the emitted photon, hc/λ_mn_, the transition probability A_mn,_ and the population of the exited state N_m_ by the following equation:

(1) $${\text{I}}_{\text{mn}}={\text{N}}_{\text{m}}{\text{A}}_{\text{mn}}\text{hc}/\lambda_{\text{mn}}$$

Assuming Boltzmann statistics, N_m_ can be expressed as:

(2) $${\text{N}}_{\text{m}}=(\text{N}/\text{Z}){\text{g}}_{\text{m}}\exp(-{\text{E}}_{\text{m}}/\text{kT})$$

where N is the total number of states, g_m_ is the degeneracy and Z is the partition function. From Eqs. 1 and 2 we can obtain:

(3) $$\ln\left(\frac{{\text{I}}_{\text{mn}}\lambda_{\text{mn}}}{{\text{A}}_{\text{mn}}{\text{g}}_{\text{m}}}\right)=\ln\left(\frac{\text{Nhc}}{\text{Z}}\right)-\frac{{\text{E}}_{\text{m}}}{{\text{kT}}_{\text{e}}}$$

It is evident that Equation (2) shows a linear dependence of the left side of the equation from the level energy E_m_. The Boltzman plot method consists then in plotting Equation (2) for several spectral emission lines belonging to the same chemical species and perform a linear fit. As evident from Equation (2), the electron temperature T_e_ is immediately inferred from the slope of the linear fit.

The electron temperature can be also estimated by use of the intensity ratio of just a pair of emission lines, labeled (1) and (2) in the following equation, among those selected for the Boltzmann plot:

(4) $$\frac{\text{I}(1)}{\text{I}(2)}=\frac{\text{A}(1){\text{g}}_{\text{m}}\left(1)\lambda (2\right)}{\text{A}(2){\text{g}}_{\text{m}}\left(2)\lambda (1\right)}\exp\left[-\frac{{\text{E}}_{\text{m}}(1)-{\text{E}}_{\text{m}}(2)}{{\text{kT}}_{\text{e}}}\right]$$

Extracting T_e_ from Eq.4:

(5) $${\text{T}}_{\text{e}}=\frac{{\text{E}}_{\text{m}}(2)-{\text{E}}_{\text{m}}(1)}{\text{k}\ln\left[\frac{\text{I}(1)\text{A}(2){\text{g}}_{\text{m}}\left(2)\lambda (1\right)}{\text{I}(2)\text{A}(1){\text{g}}_{\text{m}}\left(1)\lambda (2\right)}\right]}$$

Since the emission line parameters are well known, this method is particularly useful because it does not require too many calculations and can easily be implemented in real-time measurements.

The principle of Covariance Mapping Technique (CMT) is based on the assumption that the spectrum under investigation can be considered as a sampling function for the signal coming from the detector. Under this hypothesis, if x_k_(λ_i_) is the signal recorded at wavelength λ_i_ of the k-th spectrum, the covariance matrix of the series of N spectra is given by:

(6) $${\text{C}}_{\text{ij}}=\frac{1}{\text{N}}\sum_{\text{k}=1}^{\text{N}}{\text{x}}_{\text{k}}\left(\lambda_{\text{i}}\right){\text{x}}_{\text{k}}\left(\lambda_{\text{j}}\right)-\left[\frac{1}{\text{N}}\sum_{\text{k}=1}^{\text{N}}{\text{x}}_{\text{k}}\left(\lambda_{\text{i}}\right)\right]\left[\frac{1}{\text{N}}\sum_{\text{k}=1}^{\text{N}}{\text{x}}_{\text{k}}\left(\lambda_{\text{j}}\right)\right]$$

The covariance matrix is clearly symmetric upon exchange of *i* and *j* and in our analysis, we use the normalized form of the matrix, defined by:

(7) $${\text{m}}_{\text{ij}}=\frac{{\text{C}}_{\text{ij}}}{{\left({\text{C}}_{\text{ii}}\cdot {\text{C}}_{\text{jj}}\right)}^{1/2}}$$

The value of m_ij_ lies in the range between -1 and 1, and the diagonal elements m_ii_ are equal to 1. m_ij_ = 1 indicates correlated pairs of chemical species, whereas m_ij_ = -1 indicates anti-correlated lines of the spectrum. In this condition each peak of the spectrum can be viewed as a region of several points which can or cannot be correlated with other regions of the spectrum itself.

Correlated or anti-correlated regions are the result of correlated or anti-correlated light emissions which are distinguishing features of the chemical species present in the plasma plume. The main feature of the CMT resides in its reliability in extracting the information in case of very weak lines, provided that change in intensity can be statistically correlated to the variation of the intensity of some other lines. The CMT is thus particularly powerful to evidence the contribution of weak spectral signals which carry information but are characterized by a small value of the signal/noise ratios.

**Figure 2.**
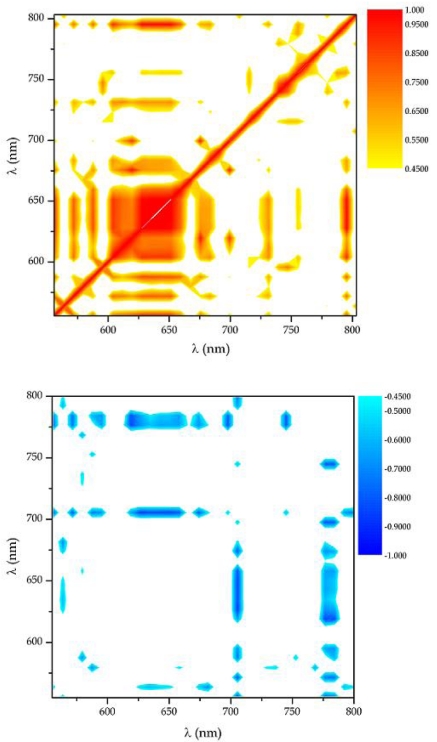
Correlation (top) and anti-correlation (bottom) maps for Al-Mg aluminium alloys.

In Figure 2 two samples of the correlation and anti correlation maps in the case of laser welding of Al-Mg Aluminium alloy are shown. A positive correlation value between the intensity of emission coming from two different species, identified by the emission wavelength and seen as reddish zones in the upper map, indicates that the evolution of their concentration in the plasma plume is the consequence of a process that has similar features for both the investigated species. On the other hand, a negative correlation means that the two species are formed by competing processes. In case of non-correlation, the species evolve through unrelated mechanisms and no useful information can be obtained. In our analysis, only the values of m_ij_ corresponding to a confidence level above 95% were considered. The threshold value fulfilling this criterion depends only on the number of spectra used in the covariance matrix evaluation.

## 3. Experimental Procedure and Results

Several welding trials have been carried out in recent years to check whether the electron temperature and the correlation coefficient signals can be a valuable source of information for the development of a real-time monitoring sensor. The trails were conducted by using a high power CO_2_ laser with a maximum output power of 2.5 kW. The plasma optical emission was collected by a quartz collimator of 6 mm focal length, and the light then transmitted to a PC interfaced miniaturized spectrometer. The spectral range investigated was 390-800 nm with a spectral resolution of 0.3 nm. The final result of our tests is an optical sensor that embeds a fiber-coupled miniature spectrometer with a dynamic spectral range from 390 nm to 580 nm and a resolution of 0.3 nm, which performs a real-time data acquisition and spectral analysis integrating the information given by the two spectroscopic techniques previously described.

The electron temperature was monitored for different chemical species inside the plasma plume, chosen according to the metal which is being welded finding a clear correlation between the temperature mean value, its standard deviation and the quality of the welded joints. This information was used to optimize the welding parameters. We also showed that local perturbations and/or variations of the temperature value are related to the occurrence of weld defects. The defects most sensitive for this technique are: i) lack of penetration; ii) weld disruptions; iii) crater formation and also iv) seam oxidation.

We created a defect detection algorithm by setting a reference value and two adjustable error thresholds for the electron temperature. The reference signal was computed during a self-learning procedure based on preliminary acquisition of sound welds performed in similar operating conditions. The upper and lower error thresholds were defined by adding or subtracting an adjustable fraction of the average standard deviation of sample signals, so setting the desired sensitivity of the monitoring system.

**Figure 3.**
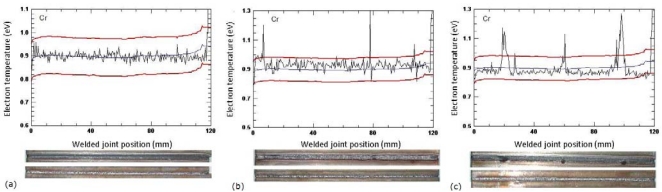
Electron temperature signals along the welded joints in presence of (a) a defect free weld (b) laser power variations and (b) crater formation.

Figure 3 shows the electron temperature signals correlated to the welded joints position in three different welding conditions; in (a) the electron temperature for defect-free weld is shown. It is evident that the electron temperature value is always in between the chosen thresholds. In (b) and (c), on the other hand, it is visible a clear increase of the electron temperature value for variable laser power and in presence of craters in three different points of the joints.

For what concerns the second technique used in the sensor, we have successfully used CMT to relate the dynamics of the chemical species present in the plasma plume to the characteristics of the welded joint. In case of laser welding of Al-Mg alloys, we have demonstrated that when there is either a collapse of the keyhole, due to the incorrect setting of the welding speed, or a loss of Magnesium, due to an inappropriate choice of the laser power, there is a marked change in the correlation signal between Al and Mg. The signal can flip from positive to negative correlation or viceversa [19, 20]. Needless to say the collapse of the keyhole causes always a defect in the welded joint.

**Figure 4.**
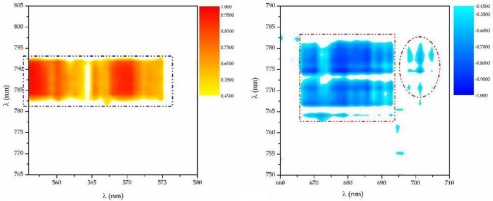
Correlation and anti-correlation map for Mg and Al for effective and ineffective shielding gas condition.

Our findings have been confirmed by EDX analysis of the metallographic cross section of the allegedly defective welded joints. Moreover, we were able to correlate the influence of the welding speed on the loss of alloying elements by monitoring the correlation/anti-correlation between environmental gases (N and O) and Mg, which is a ligand of AA5083. As shown in Figure 4, we observed an high anti-correlation between Mg and Al, in cases of ineffective shielding gas, consistently with the post-process EDX analyses performed on the welded joints and an high correlation between these two species for effective gas shielding conditions.

The measure of the correlation (anti-correlation) signal for a selected couple of lines allows to overcome the main drawback for the use of CMT in real-time sensors, that is the long acquisition and computation time requested for building the Covariance Map of the whole spectrum. By selecting only pairs of lines only a few number of the Covariance Matrix have to be evaluated paving the road towards the use of such a technique for real-time sensors [20].

Field tests have been carried out on an industrial production line of laser welded stainless steel tubes. The laser-metal interaction zone was shielded by argon flux. In this case Mn(I), Fe(I) and Cr(I) spectral lines were used for the acquisition of the electron temperature as well as for the correlation signals. The tested sensor was able of monitoring simultaneously the temporal evolution of the plasma plume and of the correlation coefficient during the welding process. For the covariance analyses, we used a sliding window of N=10 consecutive spectra. Together with the process speed N determines the maximum achievable spatial resolution of the correlation signals and also influences the value m_ij_ corresponding to the confidence level of 95% which was selected as threshold for the joint quality evaluation procedure.

The sensor was then calibrated with optimized welding parameters and its response to the occurrence of welds was also verified. Such procedure helped in tuning the sensitivity of the sensor algorithm. The final result we obtained was that the sensor has proven is capability in detecting the most important welding defects such as lack of weld penetration, bead surface oxidation (due to ineffective gas shielding), gaseous inclusions (due to surface pollution for inappropriate choice of the main operating parameters, as shielding gas flux, welding speed, laser power).

**Figure 5.**
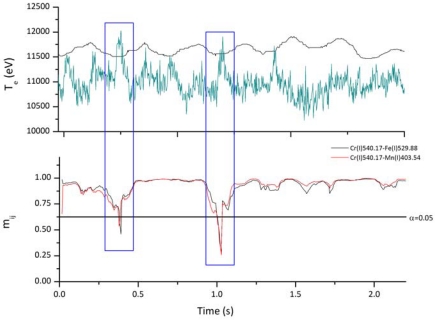
Cr(I) electron temperature and correlation signals for variable welding speed.

Figure 5 illustrates the electron temperature and correlation signals evolution in presence of variable welding speed. It is evident that instabilities in the molten pool as well as in the plasma plume are clearly detected by the sensor. In fact both the electron temperature and the correlation signal values exceed the threshold values.

It is worth mentioning that one of the main advantages of our sensor lies in the relatively easy procedure for the calibration which allows any operator to optimize the values of the main process parameters, like the welding speed or the laser power to obtain sound welded pipes.

## 4. Conclusions

In this paper we have briefly reviewed the state of art of optical sensors for laser welding process. Several techniques demonstrate the effectiveness of using optical signals for sensitive, selective and real time defect detection. We also reported on the development of a spectroscopic sensor, patented and now available on the market, based on the acquisition of the optical spectra emitted from the laser generated plasma plume. From the optical spectra the sensor evaluates the electron temperature and analyses the correlation between selected pairs of spectral lines. All the calculations are performed in real-time.

Compared to other optical sensors, the main advantage of this system is that it has a great flexibility upon variation of the welding metal or the joint geometries. In fact once the chemical composition of the alloy is known and the relevant plasma emission lines are identified, only a minor calibration of the software settings is necessary. Provided that the emission line parameters are known, this method is particularly user friendly because it does not require too many calculations and can be easily implemented for real-time temperature monitoring.

## Figures and Tables

**Figure 1.**
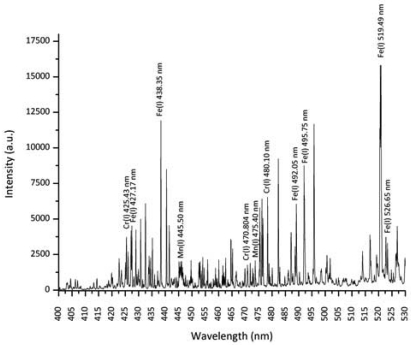
Plasma optical emission in the range 400-530 nm with a resolution of 0.07 nm, obtained during CO_2_ laser welding of AISI 304 stainless steel.
